# The Association Between Myasthenia Gravis and Higher Extrathymic Cancer Risk

**DOI:** 10.1002/brb3.70143

**Published:** 2025-01-19

**Authors:** Mohamed Khateb, Mai Abu Zant, Alaa Bsoul, Tomer Karny, David Yarnitsky, Shahar Shelly

**Affiliations:** ^1^ Department of Neurology Rambam Medical Center Haifa Israel; ^2^ Department of Data Analysis and Information Systems Rambam Medical Center Haifa Israel; ^3^ Rappaport Faculty of Medicine Technion‐Israel Institute of Technology Haifa Israel; ^4^ Department of Neurology Mayo Clinic Rochester Minnesota USA

**Keywords:** cancer, epidemiology, myasthenia gravis, paraneoplastic, seropositive

## Abstract

**Background:**

Myasthenia gravis (MG) is strongly associated with thymic tumors, but whether it is also associated with extrathymic cancers is debatable or whether MG can be considered a paraneoplastic disorder for extrathymic cancers.

**Methods:**

This is a retrospective analysis of the MG cohort for 23 years' time (January 2000 to May 2023), extracting cancer rates with clinical, electrophysiological, and biochemical cancer associations and the effect of chronic medications.

**Results:**

We identified 436 patients with MG and 3924 controls. The median age at symptom onset was 64 (5–93 years) for males and 54 (1–87 years) for females. MG symptoms at onset were recorded as ocular (60%), strictly bulbar (10%), or generalized (23%). Extrathymic cancer was found in 32% of MG patients. In 3%, thymic and extrathymic cancers co‐occurred. Compared to controls, neurology (12.2%, 159/1308), internal medicine (24.4%, 319/1308), or rheumatology (12%, 157/1308), MG patients had significantly higher rates of extrathymic cancers (*p* < 0.001). Compared to the rheumatology group, the cancer relative risk of 2.97, CI = 2.5–3.4. Furthermore, the prevalence of extrathymic cancers was significantly increased within the paraneoplastic time window, defined as ±5 years from cancer diagnosis to myasthenia onset (*p* < 0.01).

**Conclusion:**

MG was significantly associated with an increased risk of extrathymic cancers, particularly within the paraneoplastic time window. These findings suggest that MG might potentially behave as a paraneoplastic disorder.

## Graphical abstract

We retrospectively investigated extrathymic cancer incidence in the myasthenia gravis group compared to three age‐ and sex‐matched control groups, neurological, rheumatological, and internal medicine patients. Further questions, such as incidence within the paraneoplastic time window and possible risk factors for this increased cancer incidence in the MG group, were also investigated.

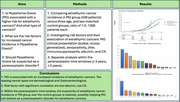

## 1 Introduction

While myasthenia gravis (MG) is known to be associated with thymic tumors, its association with extrathymic cancers is debatable. Currently, a paraneoplastic extrathymic status for MG cannot be established due to a lack of sufficient supporting data. Paraneoplastic neurological syndromes were previously defined according to three main parameters: the increased incidence of cancers in patients with neurological syndromes, the unique temporal pattern of cancer incidence within a specific time (up to 5 years before or after), and the existence of paraneoplastic antibodies (Elrington et al. 1991; Graus et al. 2004; Shah et al. 2022).

Numerous publications examined the general association of MG and extrathymic cancers, questioning such a possibility. Extrathymic cancer rates were highly variable, ranging from 8.9% in an Italian study to 25% in a US‐based study in patients who were diagnosed with MG (Citterio et al. 2009; Shelly et al. 2021). These studies could not determine a paraneoplastic mechanism of MG for extrathymic cancers due to the lack of data on the temporal relationship between MG and the reported extrathymic cancer. Moreover, they lacked relevant control groups that matched, regarding several relevant aspects, to the MG group or proper matching in systemic autoimmune state and chronic immunosuppression (Basta et al. 2014; Evoli et al. 1998; Liu et al. 2012; Sasi et al. 2022; Shaygannejad, Ghasemi, and Rajaee 2011; Slegers et al. 2022; Uğur et al. 2022; Zheng et al. 2021). However, other studies did not support the association of MG with an increased risk for cancer, showing the incidence rate for extrathymic cancer lower in the MG group (Basta et al. 2014; Evoli et al. 2004; Owe, Daltveit, and Gilhus 2006), concluding that the immunological process underlying MG does not influence the risk of extrathymic cancer (Evoli et al. 1998; Owe, Daltveit, and Gilhus 2006). An additional study that investigated the incidence of extrathymic cancers in thymoma patients showed that cancer incidence was higher in non‐MG patients compared to thymoma patients with MG (Evoli et al. 2004).

This study aimed to investigate the incidence of extrathymic cancers in patients with MG, with a particular focus on the paraneoplastic time window. Addressing the limitations of prior research, we compared our MG cohort with age‐ and sex‐matched disease control cohorts. This approach enhances the robustness of our findings by mitigating potential confounding factors and provides a more accurate assessment of the association between MG and extrathymic cancers.

## 2 Methods

### 2.1 Study Design, Ethical Considerations, and Study Cohort

This retrospective cohort study was conducted after the approval of our institutional IRB. Files of patients with a confirmed diagnosis of MG, availing medical attention at the neurological department and clinic in a solitary tertiary medical facility spanning the time frame from January 1, 2000, to May 31, 2023, were screened. MG cases diagnosed in the oncological clinics were excluded to minimize reporter bias. MG diagnosis was based on the combination of both a clinical picture of MG and a supportive electrodiagnostic or serological (for acetylcholine receptor or muscle‐specific tyrosine kinase) testing. Exclusion criteria were as follows: (1) immune modulatory therapy before 6 months of antibody testing or (2) the use of acetylcholine esterase inhibitors within 8 h of electrodiagnostic (EDX) testing. Two‐hertz repetitive nerve stimulation (RNS) with a train of four stimuli was utilized to assess for a postsynaptic neuromuscular defect. RNS was deemed confirmatory when a physiologic pattern of decrement of > 10% was seen in the compound muscle action potential at baseline or up to 3 min post 1 min exercise in two or more motor nerves without alternative explanation. Single‐fiber EMG (SFEMG) positivity was determined utilizing quality and cutoff guidelines previously published (Chiou‐Tan and Gilchrist 2015; Padua et al. 2000). A negative serological status was not considered an exclusion criterion as part of myasthenic patients is known to be seronegative.

### 2.2 Data Compilation

Demographic and clinical data were carefully gathered from the medical dossiers of the eligible subjects. Incidence of cancer was ascertained via cross‐referencing with our hospital‐based critical vital statistics archives. A comprehensive and thorough evaluation of each case was undertaken, with experienced neurologists (M.K., A.B., M.A.Z., and S.S.) overseeing the chart review process, ensuring accuracy.

### 2.3 Cancer Association

We reviewed all instances within both the case and control groups, ensuring the substantiation of malignancy presence and specific categorization by the organ involved. The classification of cancer encompassed parameters such as the date of diagnosis or age at the time of cancer identification, the precise cancer subtype using pathology and oncological reports, and the temporal interval between myasthenia diagnosis and the diagnosis of cancer. We defined the date of MG onset as the time—0 for the analysis; when no clear date of onset was recorded (eight patients), the date of the first documented follow‐up was used. We validated our data through a combination of consistent oncological clinical surveillance and pathological verification for each cancer. The characterization of malignancies was predicated upon the primary tumor's anatomical localization, with due exclusion of premalignant skin alterations, prostate hyperplasia, gastrointestinal (GI) polyps, premalignant hematologic disturbances, and others. In situations where multiple distinct cancer incidents were detected within the study duration, priority was accorded to the cancer diagnosis closely aligned in time with the myasthenia diagnosis for the purposes of cancer association calculations.

### 2.4 Control Matching

Control group analysis was facilitated by employing the MDClone platform (MDClone Ltd., Beer Sheva, Israel). The MDClone software, seamlessly integrated into our electronic medical records system, empowers the formulation of intricate search queries and streamlines access to the complete repository of retrospective hospital data. For this study, three distinct control clusters were examined, with a specific focus on age and sex harmonization. These groups encompassed patients without diagnosis of MG, referred to either the neurological, rheumatological, or internal medicine departments within the same tertiary healthcare facility. The data extraction process was tailored to align with age and sex parameters while ensuring the exclusion of myasthenic patients. The resultant dataset was comprised of 1308 individuals from each of the control groups. Specifically, for each MG patient, three random patients exhibiting matching age and sex attributes were systematically gathered from either of the control cohorts. The rheumatological control group was designed to offer control regarding chronic autoimmune diseases and the use chronic immunosuppressant agents. The other two cohorts help in being a control for the time dispersion of the appearance of cancer in relation to that of MG.

### 2.5 End Points and Statistical Analysis

The primary end point was the presence of lifetime cancer histopathologically approved. Descriptive statistics were used to summarize the characteristics of the study population. Kaplan–Meier curves were used to estimate the probability of cancer over time in cumulative analysis curves. A two‐tailed *p* < 0.05 was considered statistically significant.

### 2.6 Data Availability Full Data Access Statements

Authors take full responsibility for the data, the analyses and interpretation, and the conduct of the research; they have full access to all the data; and that they have the right to publish all data. Anonymized data not published within this article will be made available by request from any qualified investigator.

## 3 Results

### 3.1 Patient Characteristics, EDX, and Clinical Features

We identified 436 patients with MG. The median age at symptom onset was 64 (range: 5–93 years) for males and 54 (range: 1–87 years) for females. MG symptoms at onset were recorded as ocular (60%, 260/436), strictly bulbar (10%, 45/436), or generalized (23%, 100/436) onsets, with 7% not well‐documented. Median follow‐up time was 3 years (range: 1–34 years) with an average of 5.2 years. Serological status was confirmed for review in 51% of cases. Serologically positive cases were found in 78%, of which 75.6% (168/222(were AchR and 2.7% (6/222) were positive for MuSK, with 21.6% (48/222) double seronegative. Electrophysiological testing was available for review in 67% (294/436) of patients who underwent SFEMG or RNS or both (SFEMG 99%, 291/294 or RNS 35%,102/294). RNS was abnormal in 42% (43/102), with abnormal SFEMG in 89% (259/291). The mean jitter in the abnormal group was 38.33 µs (range: 18–167 µs) among the positive patients. Chest CT was performed in 96% (420/436), with thymic mass detected in 31% (136/436). Thymectomy was carried out in 32% (140/436) of patients, and 29% of the thymic masses (39/136) were histopathologically malignant.

### 3.2 Cancer Incidence Among MG Patients

**FIGURE 1:**
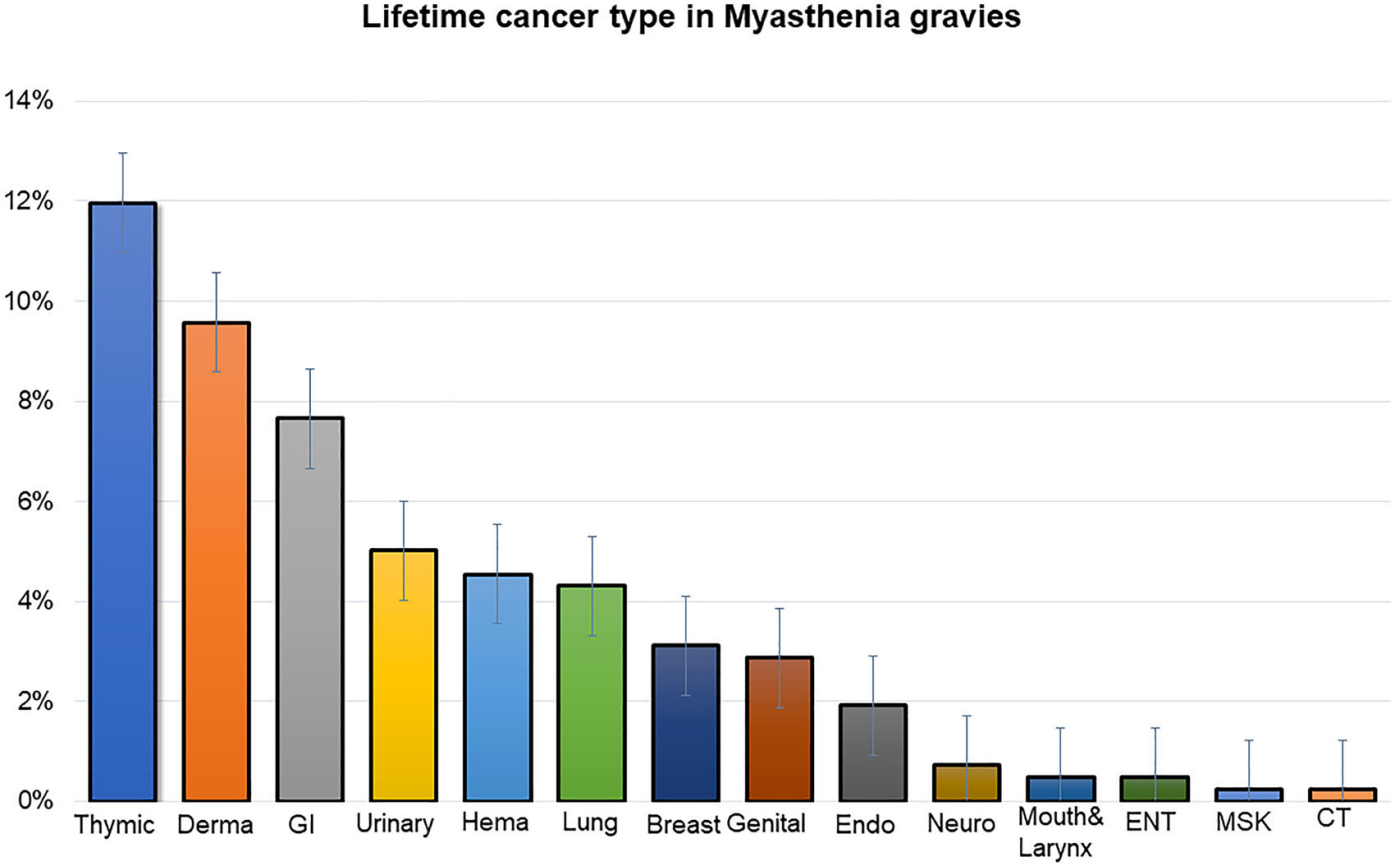
Showing lifetime cancer incidence in MG cohort: Histogram depicting the distribution of cancer subtypes among individuals with myasthenia gravis (MG). The *X*‐axis represents the percentage of patients, while the *Y*‐axis enumerates different cancer types. The most prevalent lifetime cancer types are highlighted: thymic cancers (blue): Demonstrating the highest frequency, thymic malignancies emerge as the predominant cancer subtype in MG patients, dermatological cancers (orange): The second most common cancer type observed, emphasizing a notable association between MG and dermatological malignancies, and gastrointestinal cancers (gray): Representing a significant proportion, gastrointestinal cancers underscore an additional facet of cancer incidence in MG. These findings contribute to a comprehensive understanding of the cancer landscape within the MG population, shedding light on potential associations.

**FIGURE 2:**
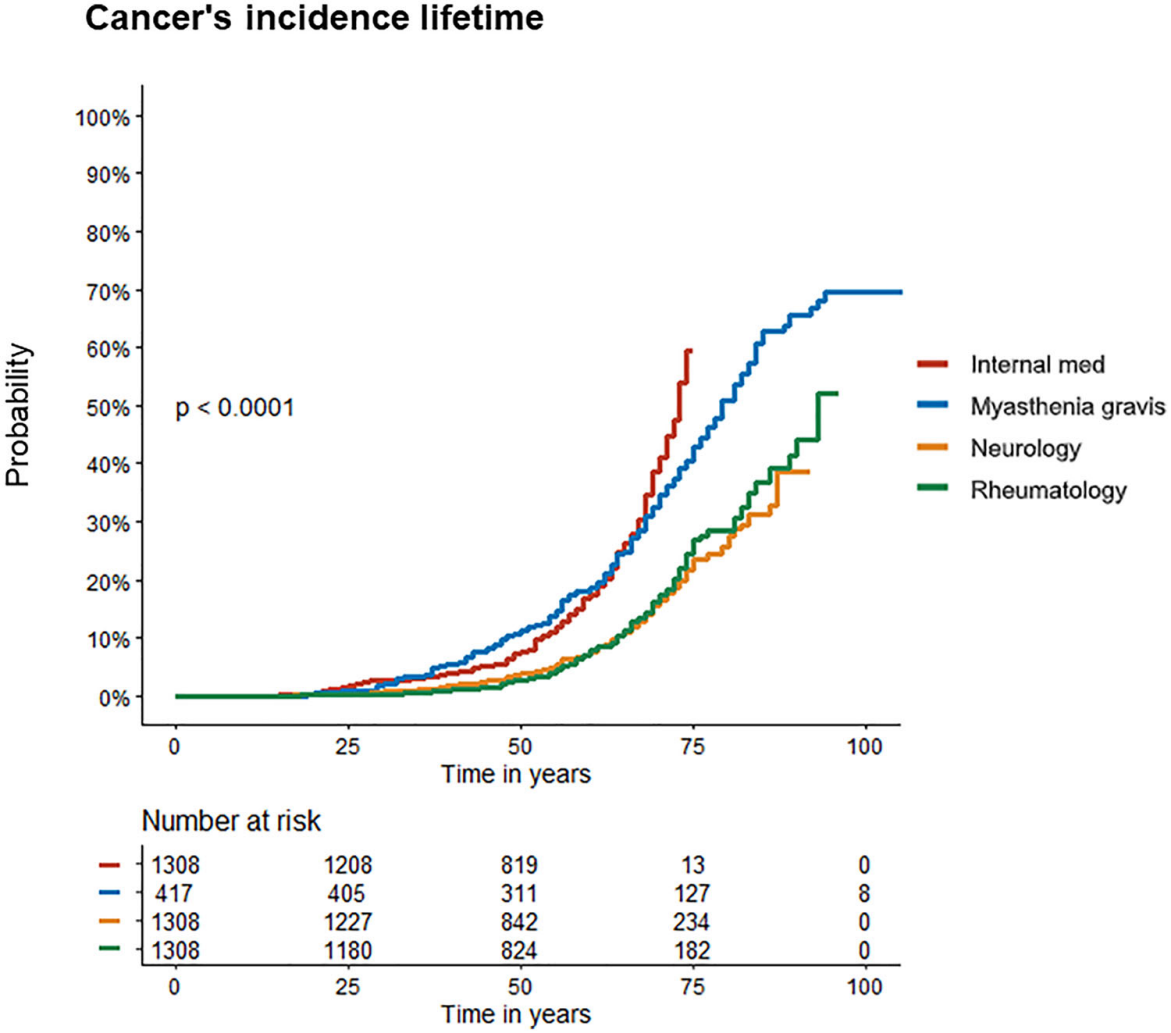
Cumulative incidence curve illustrating cancer occurrence over time in MG patients compared to control groups. Patients lacking available slides for review were excluded from this analysis. The blue curve represents the myasthenic group, showcasing the cumulative incidence of cancer cases. Notably, this group exhibits a higher frequency of cancer compared to control groups, internal medicine controls (red), demonstrates a distinct contrast in cancer incidence compared to the myasthenic group, neurology controls (orange), and rheumatology controls (green). The separation in cancer incidence between these groups becomes evident around the initiation of the disease. Below the graph, the relative number of patients at risk at specific time points provides additional comprehension of the temporal dynamics of cancer development. This analysis stresses the association between MG and increased cancer risk, presenting a nuanced perspective by comparing distinct control groups within internal medicine, neurology, and rheumatology.

Information regarding the possible occurrence of malignancy was available in 418 MG patients. Forty‐one percent of patients (172/418) had documented cancer, from which 12% (50/418) had thymic and 32% (134/418) had extrathymic cancers. In 3% (12/418) of patients, thymic and extrathymic cancers co‐occurred. Cancer prevalence is shown in Figure 1. MG patients had significantly higher rates of malignancies compared to controls, *p* < 0.0001, and compared with the internal medicine group, were twice more likely to develop cancer (OR = 2.1 CI: 1.7–2.7) as well as compared to the rheumatology group with relative risk = 2.9 (CI: 2.3–2.4) (Figure 2). Specifically, the incidence of extrathymic cancer in each of the groups was as follows: neurology (12%, 159/1308, *p* < 0.0001), internal medicine (24%, 319/1308, *p* < 0.01), or rheumatology (12%, 157/1308, *p* < 0.0001) (Table 1). Neither MG subtype nor serological status was associated with the incidence of extrathymic cancers. The incidence of cancer in ocular and bulbar MG was 32% (for each) and 31% for generalized, *p* > 0.05. Incidence was 27% (43/162) in seropositive patients (ACh‐R) compared to 35% (17/48) in seronegative patients (*p* = 0.26).

**TABLE 1:** Demography and cancer rates.

Variable^a^	Myasthenia gravis (*n* = 436)	Internal med (*n* = 1308)	Neurology‐general (*n* = 1308)	Rheumatology(*n* = 1308)	*p* value
Age at onset^b^	55.7	54.77	57.97	56.33	IM: 0.11 Neuro: 0.09 Rheu: 0.18
Sex (M:F)	Males: 47.01% (205/436) Females: 52.98% (231/436)	Males: 45.41% (594/1308) Females: 54.71% (714/1308)	Males: 46.02% (602/1308) Females: 53.97% (706/1308)	Males: 47.55% (622/1308) Females: 52.47% (686/1308)	IM: 0.13 Neuro: 0.7 Rheu: 0.78
ET cancer lifetime	32.06% (134/418)	24.39% (319/1308)	12.16% (159/1308)	12% (157/1308)	*p* < 0.01 for all the groups
Time from onset (MG)/matching date (controls) to ET cancer (years)	1 (range: ‐−27 to 43).	2 (range: −1 to 22)	5 (range: −1 to 22)	5 (range: 0–23)	*p* = 0.42 (MG vs. IM) *p* = 0.007 (MG vs. neuro) *p* = 0.008 (MG vs. rhuema)
ET cancer in ±3 years	14.35% (60/418)	13.45% (176/1308)	5.2% (68/1308)	4.28% (56/1308)	*p* < 0.01 (MG vs. all the groups)
ET cancer in ±5 years	21.29% (89/418)	16.59% (217/1308)	6.19% (81/1308)	6.19% (81/1308)	*p* < 0.01 (MG vs. all the groups)
Most common extrathymic cancers	Dermatological, gastrointestinal, urinary	Hemato‐oncologic, breast, gastrointestinal	Neurological, gastrointestinal, urinary	Urinary, hemato‐oncologic, breast	Not relevant

Abbreviations: ET = extrathymic.

^a^ All times are presented in median years.

^b^ When diagnosis age was not available, the closest visit was used. In the control groups, this was the age of matching.

### 3.3 Cancer's Distribution and Time to Cancer Diagnosis

The most common cancers in our MG cancer cohort were dermatological and GI related (29.9% and 23.9%, respectively, Figure 1). The average MG‐to‐cancer diagnosis time (in years) was < 1.5 months for thymic cancer, as in most of these cases, the thymic cancer was diagnosed within the same year as the MG symptoms onset (90%, 45/50). In the leading four types of extrathymic cancer, the average time was 4 years: 6.5 years for dermatological, 4 years for GI, 2.3 years for renal and urinary, and 3.8 years for hematologic. Pre‐ and post‐MG diagnosis comparison resulted in 61% of extrathymic cases diagnosed post‐MG onset, while 39% were diagnosed pre‐MG onset, *p* = 0.13. The incidence of the leading extrathymic cancer types did not significantly change between the time windows of pre‐ and post‐MG onset.

### 3.4 MG and the Paraneoplastic Time Window

**FIGURE 3:**
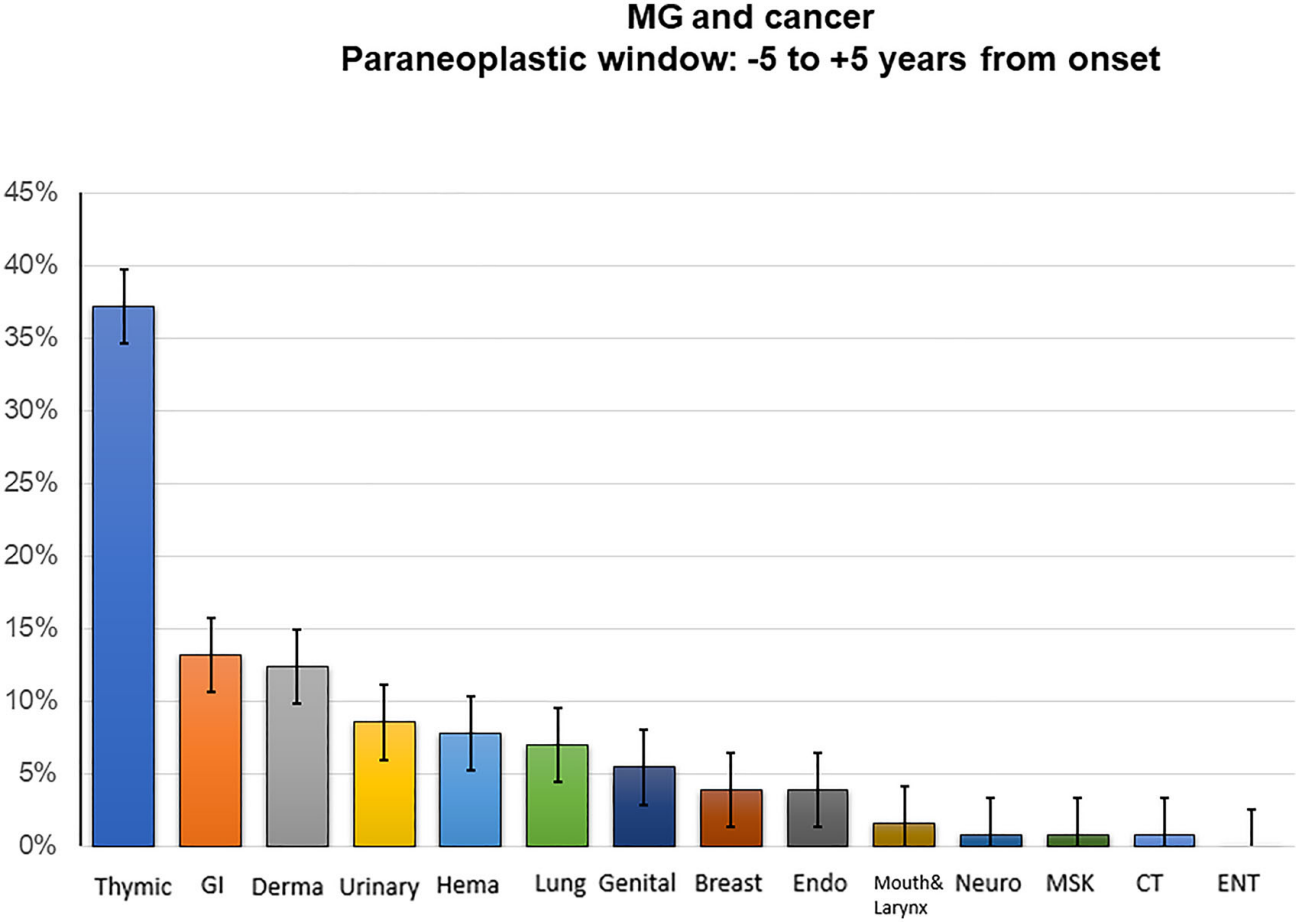
Cancer incidence in MG cohort in 5 years: Histogram depicting the distribution of cancer subtypes among individuals with MG at first before and after 5 years from symptoms. The *X*‐axis represents the percentage of patients, while the *Y*‐axis enumerates different cancer types. The most prevalent lifetime cancer types are highlighted: thymic cancers (blue): These demonstrate the highest frequency and gastrointestinal cancers (orange): The second most common cancer type observed. These findings contribute to a comprehensive understanding of the cancer landscape within the MG as a paraneoplastic disease.

As expected, the narrower the time window of analysis bordering MG onset, the higher the ratio of the thymic cancers. At ±5 years from MG symptoms onset, thymic cancer incidence was as high as 37% (48/129). This ratio further increased within ±3 years from onset; 47% (47/99) versus the incidence of 29% (50/172) regardless of the time from onset, *p* < 0.01. Within this window of ±5 years, extra thymic cancers were found in 21.3% (89/418), significantly higher than cancer incidence in the three control groups, *p* < 0.01 (Table 1). Cancer subtypes' distribution in this time window is shown in Figure 3. GI and dermatologic cancers also persisted as the leading two cancers: 13.2%, 17/129 and 12.4%, 16/129, respectively. Notably, the rising in cancer incidence in MG, compared to the control groups, is retained at the paraneoplastic time window compared to other time windows (Table 1).

### 3.5 Cancer's Pre‐ and Post‐Myasthenia Pharmacotherapy

Medication types and extrathymic cancer association were analyzed. Patients on rituximab (38/436) showed cancer incidence of 42% (16/38), 37% (14/38) of whom had cancers post‐MG onset. In these 14 patients, the leading cancer was hematologic, followed by thymic: 36% and 28%, respectively. Azathioprine‐treated patients (195/436) showed cancer occurrence of 46% (90/195), with 38% (74/195) post‐MG onset. The leading extrathymic cancer was dermatologic (26%). In the IVIG‐treated group, 33% (25/76) of the patients treated with maintenance IVIG had anytime cancers, of which 25% (19/76) were diagnosed after MG onset. For plasma exchange and steroids, cancer incidence after MG diagnosis was equal to 33% (28/85) and 35% (88/254) of patients, respectively. The IVIG‐treated group had lower cancer incidence versus azathioprine and almost reached significance when compared with prednisone (*p* = 0.03 and 0.06, respectively). The cancer distribution of each medication is shown in Table 2.

**TABLE 2:** Myasthenia gravis patient's variables and biomarkers for cancer diagnosis.

Variable	Patients with any cancer, anytime	Patients without any cancer	Cancers after MG onset	Extrathymic cancer after MG onset	*p* value
Aza (*n* = 195)	90 (46)	105 (54)	74 (38)	55 (28)	*p* = 0.03 for IVIG vs. aza *p* = 0.06 for IVIG vs. prednisone All other: *p* > 0.05
Rituximab (*n* = 38)	16 (42)	22 (58)	14 (37)	11 (29)	
Prednisone (*n* = 254)	114 (45)	140 (55)	88 (35)	61 (24)	
IVIG (*n* = 76)	25 (33)	51 (67)	19 (25)	13 (17)	
PE (*n* = 85)	33 (39)	52 (61)	28 (33)	16 (19)	
CK—max (U/L)	156.26 [209]	370 [2764]	NA	161.4 [216]	0.36
CK—min (U/L)	62 [62]	76.87 [88]	NA	59.5 [64.7]	0.05
CK—avg (U/L)	96.55 [83]	169.5 [786]	NA	96.2 [85.12]	0.27
Albumin—max (g/dL)	4.06 [0.61]	4 [0.65]	NA	4 [0.63]	0.95
Albumin—min (g/dL)	3.14 [0.84]	3.38 [0.81]	NA	3.06 [0.82]	0.001
Albumin—avg (g/dL)	3.56 [0.67]	3.69 [0.66]	NA	3.5 [0.66]	0.01
Jitter (µs)	37.92 [24.4]	33.2 [20.72]	NA	36.1 [17.7]	0.4

Abbreviations: Aza = Azathioprine, CK = creatine kinase, IVIG = intravenous immunoglobulins, PE = plasma exchange.

### 3.6 Cancer Associations

We investigated whether clinical, biochemical, serological, or electrophysiological parameters may have had a possible correlation with the occurrence of extrathymic cancers. Neither the MG subtype nor electrophysiological parameters were associated with extrathymic cancer occurrence (Table 2). Seropositive MG was mainly for AchR. In this group, the incidence of extrathymic cancer was 26.54% (43/162). This ratio was lower than the incidence of extrathymic cancer in the seronegative group, 35.42% (17/48), yet not reaching statistical significance (*p* = 0.26). Six patients were anti‐MuSK antibody positive, none of these had extrathymic cancer.

Thymectomy was not associated with higher incidence of extrathymic cancer. Thymectomy patients showed 16% (22/140) incidence of cancer, compared to 18% (49/280) without thymectomy, *p *> 0.05. For this analysis, only anterograde cancers appearing after MG onset were counted. Interestingly, low albumin and creatinine kinase level were correlated with a higher rate of cancer (Table 2).

## 4 Discussion

In this comprehensive study conducted at a single center, we explored the incidence of extrathymic cancers among 436 MG patients from 2000 to 2023. Extrathymic cancer incidence was significantly higher in the MG group compared to controls (*n* = 3924). Our study stands out due to the inclusion of age‐ and sex‐matched control groups from similar demographic backgrounds, all of whom received care within the same healthcare system and underwent similar screening protocols. Notably, the inclusion of a rheumatologic control group, consisting of patients with similar autoimmune conditions treated with chronic immunosuppression, enhances our ability to assess causality and reduce bias. This approach allows for a more accurate evaluation of whether MG contributes to an increased incidence of extrathymic cancers.

While MG is associated with thymoma in up to 20%–15% of all MG patients (Rezania et al. 2012), its association with extrathymic malignancies remains a matter of debate (Levin et al. 2005; Spillane, Beeson, and Kullmann 2010). Variable risk of extrathymic cancer among MG patients was reported, ranging from low values of 2.6% up to 22.4% (Citterio et al. 2009; Levin et al. 2005; Tanovska et al. 2018; Verwijst, Westerberg, and Punga 2021; Wakata et al. 2006). In addition, a large Taiwanese national sample size study with 2614 MG patients showed an increased risk of extrathymic malignancies incidence of 1.38 (*p* < 0.01) compared to a control cohort for an 8‐year follow‐up period (Liu et al. 2012). This cohort had a control group that was matched regarding age, sex, and other comorbidities, which is alone insufficient to normalize two other important factors that may influence the results, chronic autoimmune state and the use of immunosuppressant agents in MG patients. No statistically significant relationship was found between MG and any specific malignancy. In our cohort, we have compared extrathymic cancer rates adjusted for age and sex in MG versus disease controls. About 41% of MG patients were documented to develop any cancer, of which a substantial 32% experienced extrathymic cancers before or after MG onset. This rate of extrathymic cancer incidence is higher than any of the previously reported rates in MG. These differences in cancer incidence between our MG cohort and previous studies may arise from many factors. First, we did not rely on registries but performed a manual review of each of the patient's electronic charts. Other reasons might be related to demographic and population differences. This rate was higher compared to all the control groups achieving the primary end point of the study (Figure 2).

The observed rise in the incidence of extrathymic cancers alone does not suffice to classify MG as a paraneoplastic disorder. To address this, we focused on analyzing cancer incidence within a critical paraneoplastic time window—spanning 5 years before and after the onset of MG (Graus et al. 2004). Within this time frame, the heightened incidence of extrathymic cancers, in comparison to control groups, persists. Consequently, despite all the limitations of a retrospective study, our temporal analysis supports the hypothesis that MG could act as a paraneoplastic disorder for extrathymic cancers. Yet, this conclusion cannot be confidently stated in a retrospective study. The third important component of a paraneoplastic disorder is the existence of antibodies. Various antibodies were correlated with other paraneoplastic neurological syndromes, and some were previously erroneously correlated with cancer in myasthenia (Shelly et al. 2021). However, none of these antibodies are being routinely screened when evaluating MG patients, thus, restricting our ability to investigate this component.

The exact pathomechanism of developing extrathymic cancers in MG is obscure and may be influenced by many MG‐related and nonrelated factors. Autoimmunity is one of these factors, which is not specific to MG. Incidence of cancer was shown to be increased in many other autoimmune diseases, for example, high incidences of hematological malignancies, mainly non‐Hodgkin lymphoma, were reported in patients with rheumatoid arthritis, primary Sjögren syndrome, and systemic lupus erythematosus (SLE) based on meta‐analysis, case reports, and a few cohort studies (Kassan et al. 1978; Pettersson et al. 1992; Prior 1985). Other studies, however, were not conclusive (Cibere, Sibley, and Haga 2001; Kauppi, Pukkala, and Isomäki 1997).

Another possibility affecting MG's relationship with extrathymic cancers is the chronic use of immunosuppressants. Immunosuppressant impact on cancer risk remains uncertain. Theoretically, chronic immunosuppression is correlated with pro‐oncogenic state (Confavreux et al. 1996; Jiyad et al. 2016; Pasternak et al. 2013). The rheumatological control group was designed to address this issue of chronic immunosuppressants. The finding that MG patients using immunosuppressants had more extrathymic cancers than rheumatological patients also receiving immunosuppressants supports the understanding of an increased prevalence of extrathymic cancer in MG, which is probably not related to the chronic autoimmune state or the use of immunosuppressants.

Identifying biomarkers and risk factors for extrathymic cancer in MG patients is crucial for early detection and better management strategies. As per cancer, age is one of the most important risk factors (Liu et al. 2012). As we age, the susceptibility to carcinogenesis is expected to increase. As a result, the effect of factors such as chronic inflammation or immunosuppression will also rise with age. Considering this important linkage with age, we matched this parameter between the MG and the control groups. In our cohort, there was no significant relationship between the incidence of cancer and the subtypes of MG by clinical or serological status or whether patients had undergone thymectomy. The latter is at odds with a recently published article studying long‐term follow‐up for patients after thymectomy and showing a higher incidence of cancers in patients after thymectomy (Kooshesh et al. 2023). This discrepancy may be due to several factors including different populations of patients (they had more cardiac patients instead of solely MG patients), longer follow‐up time, and larger number compared to our study.

Our study faces several limitations inherent to its retrospective case‐control design, including potential reporter bias stemming from the use of historical, patient self‐reported data. Further limitations arise from the disparity in immunological profiles and chronic medication use between the MG patients and the control groups, particularly the rheumatologic group, as well as the absence of data on paraneoplastic antibodies. An additional limitation is the effect of chest CT workup in MG patients on increased cancer detection rates in this MG group. Despite these challenges, the study's robustness is enhanced by employing three disease control groups, which help mitigate epidemiological outliers that could influence disparities in healthcare access and utilization. In addition, the study benefits from an extensive follow‐up period, accompanied by detailed medical histories, bolstering its strength and the reliability of its findings.

In conclusion, by using multiple disease control groups, our study provides a comprehensive pattern of extrathymic cancer prevalence in MG patients. Extrathymic malignancies were higher in MG compared to controls. This rise in extrathymic cancers is probably not due to chronic inflammatory state or secondary to immunosuppressive treatments decreasing the natural surveillance arm of the immune system. Besides the controversy of whether MG can be a paraneoplastic disorder for extrathymic cancers, additional important questions remain open. Whether this increase in extrathymic cancer is simply due to coincidence, thymic removal, environmental, or genetic factors predisposing for both the MG and cancer are unclear and require further research.

## Author Contributions

**Mohamed Khateb**: conceptualization, investigation, writing–original draft, methodology, validation, visualization, writing–review and editing, formal analysis, project administration, data curation. **Mai Abu Zant**: methodology, validation, formal analysis, data curation. **Alaa Bsoul**: methodology, validation, formal analysis, data curation. **Tomer Karny**: methodology, software, formal analysis, data curation. **David Yarnitsky**: conceptualization, supervision, project administration, writing–original draft, writing–review and editing, investigation. **Shahar Shelly**: conceptualization, investigation, writing–original draft, methodology, writing–review and editing, software, project administration, supervision.

## Ethics Statement

We confirm that we have read the journal's position on issues involved in ethical publication and affirm that this report is consistent with those guidelines. Approval for this study was obtained by the institutional review board at the Rambam Medical Centre, and all patients provided written consent to participate.

## Conflicts of Interest

The authors declare no conflicts of interest.

### Peer Review

The peer review history for this article is available at https://publons.com/publon/10.1002/brb3.70143.

## Data Availability

The data that support the findings of this study are available on request from the corresponding author. The data are not publicly available due to privacy or ethical restrictions.
